# Integrated Use of Biomarkers (O : N Ratio and Acetylcholinesterase Inhibition) on *Aulacomya ater* (Molina, 1782) (Bivalvia: Mytilidae) as a Criteria for Effects of Organophosphate Pesticide Exposition

**DOI:** 10.1155/2012/951568

**Published:** 2012-04-24

**Authors:** Eduardo Führer, Anny Rudolph, Claudio Espinoza, Rodrigo Díaz, Marisol Gajardo, Nuria Camaño

**Affiliations:** ^1^Centro Regional de Estudios Ambientales (CREA), Universidad Católica de la Santísima Concepción, P.O. Box 297, Concepción, Chile; ^2^Departamento de Química Ambiental, Facultad de Ciencias, Universidad Católica de la Santísima Concepción, Concepción 4090541, Chile

## Abstract

The effect of residual concentrations of organophosphate pesticide chlorpyrifos (Lorsban 4E) on the activity of the acetylcholinesterase enzyme and oxygen : nitrogen ratio in the mussel *Aulacomya ater* was analyzed. Toxicity tests show a sensitivity to the pesticide in the bivalve estimated at 16 *μ*g L^−1^ (LC_50−96 hours_). Concentrations between 0.2 and 1.61 *μ*g L^−1^ were able to inhibit significantly the AChE activity, and concentrations between 0.8 and 1.61 *μ*g L^−1^ stimulate ammonia excretion and decrease oxygen : ammonia-N (O : N) ratio, with respect to the control group. *A. ater* proved to be a species sensitive to pesticide exposure and easy to handle in lab conditions. Thus, it is recommended as a bioindicator for use in programs of environmental alertness in the Eastern South Pacific coastal zone.

## 1. Introduction

For decades, residuals of several compounds of anthropogenic origin have entered the aquatic systems, such as heavy metals, pesticides made from a range of nonnatural compounds, and other synthetic organic compounds [1]. Among these, the organophosphate insecticides have been widely used in the latter years, because of their higher level of biodegradability compared with their predecessors, the organochlorinated pesticides. Therefore, it is important to understand the occurrence of pesticides in aquatic ecosystems and their potential impact [2].

Marine bivalves like mussels are extensively used as biological indicators with the aim of quantifying the potential effects of xenobiotics, as filter feeders are able to accumulate a wide range of xenobiotics in their tissues [3]. One way to quantify the possible effects is through biomarkers which have proved a useful tool for assessing the deleterious effects of pesticides in water bodies [4]. One of these biomarkers is the quantification of the inhibition of the enzyme activity acetylcholinesterase (AChE). Acetylcholine (ACh) is considered a neuroexcitatory neurotransmitter and is involved in neuromuscular stimulation and locomotion. This neurotransmitter is regulated by AChE, which is rendered inactive by hydrolysis into choline and acetate [5]. The AChE is located in neuromuscular junctions and in bivalves, and prosobranch mollusks in particular have high levels of AChE activity in the hemolymph [6].

The organophosphate pesticides (OPs) are extremely neurotoxic and proved to be effective inhibitors of AChE activity. OP pesticides generated in mussels a hyperactivity syndrome in the nerve cells, resulting in a cell disruption product of oxidative stress and inflammation [7].

The inhibition of AChE activity has been used as a specific biomarker for the presence of organophosphorus compounds [8–11].

Another biomarker used to assess stress is the quantification of oxygen-nitrogen ratio (O : N), which indicates the physiological state of the organism in this case exposed to xenobiotic [12, 13].

This biomarker is the result of the quantification division of oxygen uptake (which is reflected in the metabolic proportions of the bivalves) and the quantification of ammonia excretion (which is indicated in the use of metabolic resources such as lipids and carbohydrates). This division of both biomarkers (oxygen uptake/ammonia-N excretion) generates an index which shows the metabolic changes in the organisms and the amount of energy available in them during periods of stress produced by pesticide contamination [14].

*A. ater* (ribbed mussel) is a commercially important, sessile species, which is long-lived and a filter-feeder bivalve. Its characteristics allow it to accumulate a wide range of xenobiotics in its tissues. This bivalve presents a continuous gamete release over the year, their spawn being related to food availability. Ribbed mussel has a wide latitudinal distribution in the Eastern South Pacific (20° to 56° LS), that is, Callao in Peru to the Strait of Magallanes in Chile [15].

This study responds to the need to identify native species off the coast of the eastern South Pacific that can be used in environmental monitoring programs and to assess the feasibility of implementing the integrated use of biomarkers (AChE activity and O : N ratio) to determine the presence of possible deleterious effects of chlorpyrifos-type organophosphate pesticides. 

## 2. Materials and Methods

### 2.1. Organophosphate Pesticide

The trademark name of the chlorpyrifos organophosphate insecticide used in this study was Lorsban 4E by Dow AgroSciences Chile S.A. The insecticide composition is active ingredient: 48% of chlorpyrifos and registered emulsifiers: 52%.

### 2.2. Biological Material

Juvenile specimens of ribbed mussel were collected (49.92 ± 4.7 mm long and 16.6 ± 4.18 g. of mass) from a low intervention area in the Coliumo Bay (36°50′ S 72°55′ W). Then, they were taken to the Lenga Coast lab (36°45′ S 73°10′ W) where it continued the acclimatization in aquariums of 500 L for seven days (15±1°C;  33 ± 1  ups; 8.1 ± 5.5 mg L^−1^ dissolved oxygen; pH 8.2 ± 0.2, 14 : 10 photoperiod, microalgae mixed cropping food). In this period of acclimatization, ribbed mussels showed exposed gills and no observed valvar closing during this period. 

### 2.3. Acute Toxicity Test

Preliminary assays were carried out (i.e. LC_50–96 hours_) with the pesticide over the ribbed mussel. The assays were performed in identical conditions to the acclimatization, but without feeding. Firstly, work was carried out in a wide range of concentrations between 0 (control) and 1000 *μ*g L^−1^ (six concentrations) and then between 0 (control) and 50 *μ*g L^−1^ (five concentrations). Six individuals were used per cuvette with three replicates per concentration. The assays were static type.

### 2.4. Tissue Selection for Enzymatic Assays

For selection of the tissue for the enzymatic study, gills and hemolymph samples were extracted from the organism control group. For an enzymatic study, the gills were macerated in a homogenizer WiseStir HS-30E to 60 Hz for 10 seconds, in 1 : 1 (grams of tissue/buffer) with phosphate buffer + Triton × 100 (pH 7.4; 0.2 M) under cold conditions. The homogenized sample was centrifuged at 10.000 g, 4°C in a Eppendorf 5804 R for 20 minutes, and the supernatant was used to measure the enzymatic activity. To avoid lethal damage to the bivalve, the hemolymph extraction was performed by perforating one side of the anterior abductor muscle and the hemolymph was extracted with a 1 mL (26 G ×1/2′′) tuberculin syringe. Then it was placed in a 1.5 mL Eppendorf and centrifuged at 9000 g 4°C for a period of 10 minutes. To measure the enzymatic activity in both cases, 40 *μ*L of supernatant was used in a UV mini-1240 Shimadzu spectrophotometer by the Ellman method [16].

### 2.5. Chronic Toxicity Test

In chronic toxicity assays, tested chlorpyrifos concentrations were 0 (control) 0.20, 0.40, 0.80, and 1.61 *μ*g L^−1^, and the exposition time was 21 days. For each concentration of chlorpyrifos, there were 30 individuals, distributed in six replicates. Reexchanges of the test solution were carried out every 48 hours, adding 6 mL of a microalgae-mixed cropping food every time. Measurements of valvar closing were performed frequently in all tested concentrations during the 21 days of exposition. The results were expressed in percentage of valve closing.

### 2.6. Biomarkers

#### 2.6.1. Enzyme Activity

Hemolymph was selected for enzymatic essays to assess AChE enzyme activity. To this end, 10 individuals were selected at random per concentration. The AChE enzyme activity was measured through the Ellman method [16]. Each measurement was performed in triplicate, and the results were expressed in AChE activity (*μ*mol acetylthiocholine min^-1 ^mL hemolymph).

Quantitation of proteins in the gill homogenized was measured by the Bradford method using 5 *μ*L of the homogenized sample before being centrifuged [17].

#### 2.6.2. Oxygen Consumption

The determination of oxygen uptake was performed by respirometry in individual chambers of 200 mL at the end of the experiment, determining the concentration of dissolved oxygen through the modified Winkler method [18].

#### 2.6.3. Ammonia Excretion

Ammonia excretion was assessed by extracting 5 mL of seawater from the individual chambers through the Bower method [19].

#### 2.6.4. O : N Ratio

The results of O : N ratio were obtained by dividing individually the oxygen uptake of each ribbed mussel by the ammonia excretion results [20].

### 2.7. Statistical Analysis

The calculation of the sublethal concentrations was carried out through the Spearman Karber statistical packet. Data calculation and graphics were analyzed through the statistical program [21]. The results were determined to be significant at *P* < 0.05.

## 3. Results

### 3.1. Acute Toxicity Test

In the acute toxicity assays, during 96 hours, juvenile ribbed mussels were exposed under concentrations between 0 and 1000 *μ*g L^−1^ of Lorsban 4E organophosphate insecticide, observing over 100 *μ*g L^−1^ a 90% of mortality. However, in lower concentrations that is, 0 and 50 *μ*g  L^−1^ a level of mortality, that is, 50 and 70%, was observed. The LC_ 50–96__hours_ value was estimated at 16 *μ*g  L^−1^ of Lorsban 4E.

### 3.2. Enzyme Activity in Gills and Hemolymph

The AChE enzyme activity in the gill tissue of a ribbed mussel showed a value of average activity estimated at 61.74 ± 15.26 (*μ*mol acetylthiocholine (ACTC) min^−1 ^mg protein), and in hemolymph it was at about 140.75 ± 31.54 (*μ*mol acetylthiocholine (ACTC) min^−1 ^mL hemolymph). Thus, it was decided to work with hemolymph due to its higher AChE activity.

### 3.3. Sublethal Effects of Pesticide in AChE Enzyme Activity

**Figure 1:**
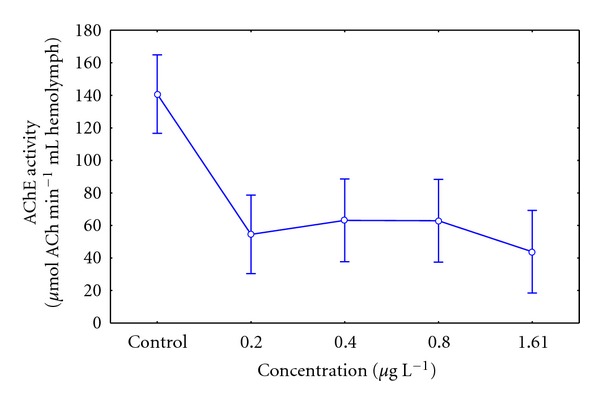
AChE activity in *Aulacomya ater* exposed to grow sublethal concentrations of organophosphate pesticide Lorsban 4E for 21 days. Mean values with standard error plotted (*statistical difference *P* < 0.05).

In the sublethal toxicity assays, after 21 days of exposure, the analyzed concentrations of Lorsban 4E, that is, 0.20–1.61 *μ*g L^−1^, showed considerable differences in the AChE enzymatic activity (Figure 1). This was observed in a significant inhibition of the AChE enzyme on each tested concentration, in relation to the individuals from the control group (*P* = 0.00007). Nevertheless, there were no major differences between the tested concentrations, that is, 0.2–1.61 *μ*g L^−1^ (*P* = 0.832); (Figure 1). 

### 3.4. Effects of Pesticide

#### 3.4.1. Oxygen Consumption

**Figure 2:**
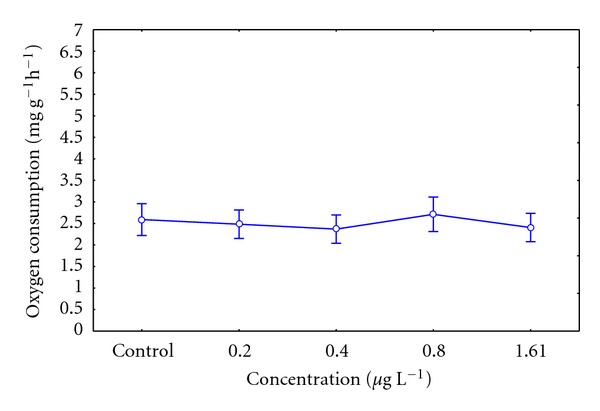
Oxygen uptake of *Aulacomya ater* in control and exposed grow sublethal concentrations of organophosphate pesticide. Mean values with standard error plotted.

The oxygen consumption did not show significant differences between the tested concentrations (i.e., 0.2 and 1.61 *μ*g L^−1^) and the control organisms (*P* = 0.6838), during 21 days of exposure to the pesticide (Figure 2).

#### 3.4.2. Ammonia Excretion

**Figure 3:**
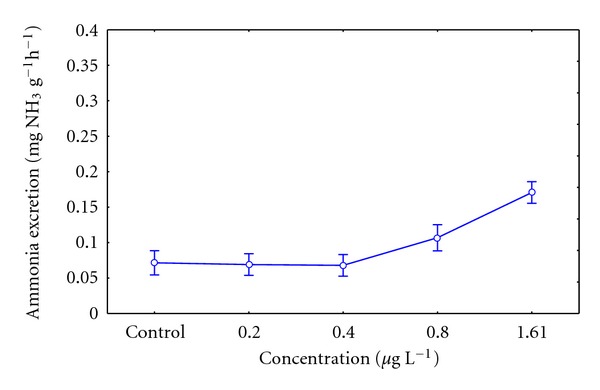
Ammonia excretion on *Aulacomya ater* in control and exposed sublethal concentrations of organophosphate pesticide. Mean values with standard error plotted (*statistical difference *P* < 0.05).

The ammonia excretion in individuals with concentrations between 0.80 and 1.61 *μ*g L^−1^ of pesticide showed average values of 0.12 ± 0.040 and 0.17 ± 0.057 (mg g^−1 ^h^−1^). These were considerably different when compared with the lower tested concentrations 0.20 and 0.40 *μ*g L^−1^ and the control group *P* = 0.0001. It shows a tendency to an increase in the ammonia excretion for concentrations higher than 0.40 *μ*g L^−1^of Lorsban 4E (Figure 3).

#### 3.4.3. O : N Ratio

**Figure 4:**
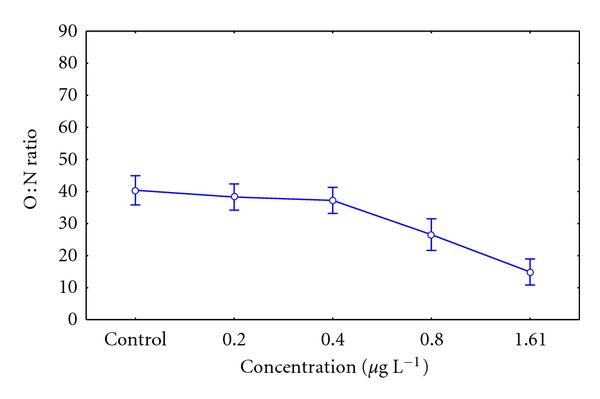
O : N ratio in *Aulacomya ater* exposed to grow sublethal concentrations of organophosphate pesticide Lorsban 4E and control group. Mean values with standard error plotted (*statistical difference *P* < 0.05).

The O : N ratio in concentrations of 0.80 and 1.61 *μ*g L^−1^of the pesticide displayed significant differences, between the lower concentrations and the control group, *P* = 0.0001 (Figure 4). However, it is observed in mussels that there is a decrease in the O : N ratio where the Lorsban 4E concentration increases in the assay, especially in the concentrations of 0.80 and 1.61 *μ*g L^−1^ of pesticide.

#### 3.4.4. Valvar Closing

Individuals in laboratory conditions (control) did not show valvar closing during the 21 days of exposure. The same behaviour was observed in individuals exposed to concentrations between 0.20 and 0.40 *μ*g L^−1^ of organophosphate pesticide. Nevertheless, in concentrations higher than 0.40 *μ*g L^−1^, the bivalves had their valve closed most of the time during the experiment (i.e., 0.80 and 1.61 *μ*g L^−1^ of organophosphate pesticide), with closing percentages between 90 and 100%, respectively.

## 4. Discussion

The valvar closing, in the present study, was the defence mechanism shown by the ribbed mussel individuals. This would help in reducing the individual's exposure to the pesticide. During most of the research, valvar closing was observed in pesticide concentrations higher than 0.8 *μ*g L^−1^. The opposite occurred in lower concentrations of pesticide tested where individuals did not show a different behaviour from the control, that is, exposed gills. This could explain the lower degree of inhibition of AChE enzyme activity in pesticide concentrations of 0.8 *μ*g L^−1^ as a significant difference was not found in the case of the 0.2 *μ*g L^−1^ concentration, that is, 25 and 33%, respectively (Figure 1).

On the type of tissue and the species analyzed by Bocquené et al. [22], variations were observed in the AChE enzyme activity. For instance, when working with bivalves, they identified the higher AChE enzyme activity in the *Mytilus edulis* gills and in the *Crassostrea gigas *oyster mantle. Also, they point out that in general the bivalves show the higher AChE enzyme activity in the gills and the abductor muscle. In this study *A. ater *showed higher AChE enzyme activity in the hemolymph extracted from the abductor muscle. Moreira et al. [6] proposed the invertebrate hemolymph as suitable biological material to be used in environmental monitoring programs, which corresponds to what was observed in this study. Moreover, it also allows a nondestructive determining of parameters such as the AChE enzyme activity in the valve.

In this study, biochemically, in all exposed individuals the AChE enzyme activity was considerably inhibited in the control group individuals, observing that the highest tested concentration, that is, 1.6 *μ*g L^−1^ reduced the AChE enzyme activity by 39% (Figure 1). Moulton et al. [23], when working on the enzyme activity in the freshwater Mytilidae's abductor muscle, concludes that percentages of inhibition higher than 30% of the AChE enzyme activity would indicate an overexposure of bivalves to organophosphate insecticides. In this study, the lower tested concentrations that is, 0.4 and 0.8 *μ*g L^−1^ reached inhibition percentages of the enzyme in relation to the control, that is, 24% and 25%, respectively. Although these values are lower than the ones estimated by Moulton et al. [23], they are close to the overexposure value of 30%. The comparison is done among individuals from the same phylogenetic branch, but living in different environments, that is, freshwater and sea water, thus it is important to highlight their similar sensibilities towards the chlorpyrifos organophosphate insecticide [24].

The oxygen consumption of ribbed mussel in the assay concentrations of insecticide, that is, 0.2 and 1.61 *μ*g L^−1^ in 21 days, did not show a defined response, which is similar to results obtained by Michaelidis et al. [25], in the bivalve *Mytilus galloprovincialis*. It is claimed that factors such as inanition, temperature, salinity, and/or pollutants would influence the nitrogen excretion in the metabolism of organisms [25]. In this study, an increase of ammonia excretion observed in juvenile ribbed mussel exposed to concentrations of 0.8 and 1.61 *μ*g L^−1^ of insecticide would be accompanied by an increase in the protein usage as a substrate during the oxidative metabolism. Similar results were observed by El-Shenawy [26], in the bivalve *Ruditapes decussates* exposed to heavy metals. 

According to Mayzaud and Conover [20], this would indicate that the oxidative metabolism of juvenile ribbed mussel organisms exposed to these concentrations may use more proteins than lipids as substrate (similar quantities of proteins and lipids provide values in the O : N ratio between 50 and 60). Moreover, they indicate that the catabolism of pure protein would be related to values between 3 and 16. In this study, the higher tested concentration of 1,61 *μ*g L^−1^of Lorsban 4E showed an average value of 14.8 ± 4.09 in the O : N ratio; thus, it is inferred that individuals exposed to high concentrations of pesticides would use only protein as an essential source of amino acids. However, they conclude that values under 10 in the O : N ratio are deleterious for the organism [20], from which it follows that the chlorpyrifos concentration 1.61 *μ*g L^−1^ tested in this study (with values close to 10 in the O : N ratio) would have induced biochemical and physiological alterations on the organisms.

## 5. Conclusions

Ribbed mussel is a species sensitive to the presence of low concentrations of organophosphate pesticide (Lorsban 4E) in the environment. It shows a significant response of inhibition of AChE activity which then promotes an increase in ammonia excretion. Therefore, this kind of mussel could be recommended for use as a biomarker in Mussel Watch Programmes in the Pacific Southwest.
